# The six-minute walk test is an excellent predictor of functional ambulation after total knee arthroplasty

**DOI:** 10.1186/1471-2474-14-145

**Published:** 2013-04-24

**Authors:** Victoria Ko, Justine Marie Naylor, Ian Andrew Harris, Jack Crosbie, Anthony ET Yeo

**Affiliations:** 1 South West Sydney Clinical School, University of New South Wales, Sydney, Australia; 2 Orthopaedics Department, Liverpool Hospital, Sydney, Australia; 3 Whitlam Orthopaedic Research Centre, Sydney, Australia; 4 Ingham Institute Applied Medical Research, Sydney, Australia; 5 School of Science and Health, University of Western Sydney, Sydney, Australia

**Keywords:** Gait, Self-reported outcomes

## Abstract

**Background:**

The Six-minute walk (6MW) and Timed-Up-and-Go (TUG) are short walk tests commonly used to evaluate functional recovery after total knee arthroplasty (TKA). However, little is known about walking capacity of TKA recipients over extended periods typical of everyday living and whether these short walk tests actually predict longer, more functional distances. Further, short walk tests only correlate moderately with patient-reported outcomes. The overarching aims of this study were to compare the performance of TKA recipients in an extended walk test to healthy age-matched controls and to determine the utility of this extended walk test as a research tool to evaluate longer term functional mobility in TKA recipients.

**Methods:**

The mobility of 32 TKA recipients one year post-surgery and 43 healthy age-matched controls were assessed using the TUG, 6MW and 30-minute walk (30MW) tests. The latter test was repeated one week later. Self-reported function was measured using the WOMAC Index and a physical activity questionnaire.

**Results:**

30MW distance was significantly shorter amongst TKA recipients (mean 2108 m [95% CI 1837 to 2381 m]; Controls 3086 m [2981 to 3191 m], P < 0.001). Test-retest repeatability was high (ICC = 0.97, TKA; 0.96, Controls). Amongst TKA recipients, the 30MW distance correlated strongly with the shorter tests (6MW, r = 0.97, P < 0.001; TUG, r = −0.82, P < 0.001). Multiple regression modeling found 6MW distance to be the only significant predictor (P < 0.001) of 30MW distance, explaining 96% of the variability. The TUG test models were moderate predictors of WOMAC function (55%) and physical activity (36%) and were stronger predictors than 6MW and 30 MW tests.

**Conclusions:**

Though TKA recipients are able to walk for 30 minutes one year post-surgery, their performance falls significantly short of age-matched norms. The 30MW test is strongly predicted by 6MW test performance, thus providing strong construct validity for the use of the 6MW test in the TKA population. Neither a short nor long walk test is a strong predictor of patient-reported function after TKA.

## Background

Maximizing functional mobility is a key goal of rehabilitation after total knee arthroplasty (TKA) surgery. The Timed-Up-and-Go (TUG) and Six-minute walk (6MW) tests are commonly used to evaluate functional recovery after TKA [1-9] as they are simple to administer and reliable [5,10]. However, for research demonstrating long-term functional recovery after TKA an extended walk test may be more useful as an outcome measure for several reasons. Firstly, little is known about walking ability beyond six minutes amongst TKA recipients. At 12 months or more after surgery, when functional performance is considered close to optimum, TKA recipients may wish to walk for longer duration for fitness or occupational needs. As such, a longer walk test can provide insight into ambulatory capacity over a more functional, everyday duration, and therefore, have greater face validity. Secondly, by extension, if a longer test approximates true functional requirements more than a shorter test, it may have a stronger correlation with self-reported behaviors than that associated with the shorter walk tests. The TUG and 6MW tests have been shown to have only moderate correlations at best with measures of self-reported function after TKA [11,12] and with physical activity in people with end-stage knee osteoarthritis [13], suggesting that self-reported function tools and timed mobility tests measure different aspects of function [4]. Consequently, both forms of assessment are considered necessary post-surgery. We contend, therefore, if an extended mobility test has greater correlation with perceived function simply because it may be more ‘functional’, then the extended walk could be a close proxy for self-reported function (or vice versa). Thirdly, a longer walk test may be useful in discriminating between individuals because it is likely that not every individual can maintain the fast paced walking achieved in the 6MW test over a longer period. It is possible that individuals who appear comparable on a short duration walk test exhibit fatigue and differences in walking endurance beyond six minutes of walking.

The use of extended walk tests has been investigated in other populations. In a study of the two-, six-and 12-minute walk tests in patients following stroke, the 12-minute walk test was observed to be the most responsive to change [14]. Further, despite high correlation between the three tests, the two-minute assessment was reported to overestimate 6 and 12 minute walking distances. The authors suggested the overestimation may be due to fatigue during the longer tests. In another study investigating patients with stable chronic airflow obstruction, a 12-minute walk test was also found to be highly correlated to six-minute and two-minute walk tests [15]. Variance of the 12-minute walk test was greater than the shorter walk tests, suggesting that the longer test was perhaps more discriminating. However, as the shorter walk tests were easier for both the patient and researcher, the authors recommended the six-minute walk as a fair compromise. Compared to other patient populations, TKA recipients may be affected by different factors which limit walking capacity, such as residual knee pain and reduced muscle strength. As such, it cannot be assumed that the results of these studies can be generalized to the TKA population.

To date, there has been no examination of the utility of a walking test beyond six minutes in the TKA population. The overarching aim of this study was to determine the utility of an extended walk test as a research tool to evaluate longer-term functional mobility in TKA recipients. The specific aims of the study were multiple: 1) to assess the performance and repeatability of the extended test in TKA recipients one year after surgery and in healthy age-matched controls; 2) to examine the correlations between the extended walk test and both the TUG and 6MW tests amongst the TKA cohort; 3) to determine the predictors of performance of the extended walk test amongst the TKA cohort; and finally, 4) to examine which of the walk tests best predicts self-reported function and physical activity. The main hypotheses to be addressed were that: TKA recipients one year post-surgery will perform significantly worse than age-matched healthy controls in an extended walk test; the shorter walk tests will not predict performance in the longer walk test, and; the longer walk test will be a stronger predictor of self-reported function and physical activity than the shorter walk tests.

## Methods

Prior to undertaking the definitive study, a pilot survey of patients awaiting knee and hip replacement was performed to determine a time parameter for an extended walk test which may be functionally relevant for this patient group. Sixty-four consecutive individuals [n = 47 awaiting TKA, mean age 68.3 (SD 10.7) years, 77% female; n = 17 awaiting total hip arthroplasty, mean age 61.5 (SD 10.9), 71% female] attending the pre-operative joint replacement education class at a public hospital completed a written survey comprising close-ended questions about current and expected 1-year walk times. At the time of the survey, 52% of participants reported a maximum walking duration of 10 minutes or less before they had to stop due to their leg symptoms. As anticipated, participants expected to be able to walk much further 1-year post surgery; 86% of participants expected to be able to walk 25 minutes non-stop, and over 50% expected to be able to walk 50 minutes non-stop one year post-surgery. From these data, we reasoned that a 30-minute walk (30MW) test was in accordance with patient expectations for functional mobility one year after TKA.

### Participants

A convenience sample of a minimum of 60 subjects (30 TKA recipients and 30 age-matched healthy controls) was planned for this study. The sample size was a compromise between gathering sufficient data for repeatability and cross-sectional analyses based on what is known from previous timed mobility trials in TKA and elderly populations (n=22 to 51) [5,11,16,17], and the need to test within the cooler, drier months (Autumn, Winter) to reduce environmental confounders.

For the TKA cohort, patients who underwent TKA 12 to 18 months prior were identified from an existing hospital database and a random sample was selected using a computer-generated sequence. The selected individuals were contacted and screened via a telephone interview. Eligible patients were invited to participate. To improve generalizability, patients with other co-morbidities and other musculoskeletal pain were not excluded, as patients who undergo TKA commonly present with multiple co-morbidities and other joint disease [18,19]. However, the following exclusion criteria were used to ensure patient safety during walk test performance: unstable angina, myocardial infarction, or cardiopulmonary, spinal or lower limb surgery in the previous six months; a recent history of uncontrolled hypertension; and inability to comprehend English.

Healthy control volunteers were recruited from the community through flyers placed in hospital grounds and through word-of-mouth. To allow comparison with a genuinely healthy population of this age group, volunteers were excluded if they had acute or chronic respiratory, cardiac, neurological and musculoskeletal disorders involving significant mobility impairment. Eligibility was determined via telephone interview.

All participants who provided written, informed consent were enrolled into the trial. The protocol and consent forms were reviewed and approved by the South Western Sydney Local Health District Human Research Ethics Committee. The characteristics of the participants including age, gender. height, and body mass index (BMI) are summarized in Table 1.

**Table 1:** Subject characteristics, WOMAC scores, and physical activity of TKA cohort and healthy controls

	** TKA (n = 32)**	**Controls (n = 43)**	** P**
Age, years (mean, 95% CI)	66.7 (64.3 to 69.2)	65.0 (62.6 to 67.5)	0.333
Gender, female	56%	56%	
Height, meters (mean, 95% CI)	1.67 (1.63 to 1.70)	1.66 (1.63 to1.68)	0.806
BMI (mean, 95% CI)	30.8 (27.6 to 34.9)	23.6 (22.2 to 27.3)	<0.001
Diagnosis of Osteoarthritis, n (%)	28 (87.5%)	0	
Number of Co-morbidities, n (median, IQR)	2 (1 to 3)	0 (0 to 1)	<0.001
WOMAC Pain, max score 50 (median, IQR)	4 (1.0 to 11.9)	0 (0 to 0)	<0.001
WOMAC Function, max score 170, higher score worse function (median, IQR)	22.8 (10.2 to 60.5)	0 (0 to 2)	<0.001
Occasions of physical activity, n (median, IQR)	7 (2 to 11)	9 (6.5 to 13.5)	<0.001
Total duration of physical activity, minutes (median, IQR)	138 (40 to 305)	390 (280 to 615)	<0.001
Use of walking aid, n	3	0	

Abbreviations:

BMI = Body mass index; CI = Confidence interval; IQR = Inter-quartile range; N = number; TKA = Total knee arthroplasty; WOMAC = Western Ontario and McMaster Universities Osteoarthritis Index.

### Experimental design

Testing took place at three outdoor sites for the convenience of the participants. An outdoor setting was used to simulate normal functional walking conditions. Well demarcated, level, paved, concrete or bitumen footpaths devoid of obstacles were chosen. Participants were tested under dry conditions with ambient temperature between 13 to 24 degrees Celsius. Time of testing was constant for each participant. Participants were instructed to wear supportive footwear and comfortable clothing.

Resting heart rate (HR) was measured (Polar F4^TM^ or F6^TM^, Polar Electro Oy, Finland) at the commencement of the testing session. As with other studies evaluating 6MW tests [16,20], HR was monitored as an indicator of participant effort. The average and maximum HRs achieved, together with the time spent with HR greater than 65% of age-predicted HR maximum, were recorded. 65% of age-predicted HR maximum was used to indicate whether a moderate level of exercise intensity had been achieved [20,21].

Four assessors used standardized instructions to administer the tests. All subjects performed walk tests in the same order. The TUG and 6MW tests were conducted first. A seated rest of at least 10 minutes was given after the 6MW test. On restoration of resting heart rate, the participant performed the 30MW test.

### Tests

#### TUG

The procedures for the TUG test was based on published protocols [22]. A standard height chair of 45cm with arm rests was placed on an outdoor level footpath, and a line 3 metres from the chair was drawn. The participant was instructed to stand up from the chair, walk to the line, turn around, walk back to the chair and sit down as quickly and safely as possible. The assessor commenced timing as the participant leaned forward to stand up, and ceased when the participant’s hips made contact with the seat to sit down. A minimum of two tests was performed for each participant with the best time used in the analysis. High repeatability of the TUG has been established in patients awaiting TKA (ICC 0.75) [23].

#### 6MW test

The procedure for the 6MW test was based on published guidelines [24]. A 25 metre section of the outdoor footpath was demarcated for this test. The participant was instructed to walk as far as possible for six minutes, up and down the demarcated footpath, pivoting to turn at the end of each lap. Timing commenced as the participant stepped over the start line. Standardized encouragement was given to the patient after each minute. The participant was instructed to stop at six minutes, or prior if they were unable to complete six minutes, and to maintain their position whilst the assessor measured the final partial lap with a trundle wheel. The use of a walking aid and standing rests were permitted. One test was performed for each participant as we did not want fatigue from completing two 6MW tests to undermine performance in the longer test. High repeatability of the 6MW test has been established in patients awaiting TKA (ICC 0.94) [23].

#### 30MW test

For the 30MW test, a 200 m section of the footpath was demarcated. A 200 m lap was chosen as it kept the frequency of turns low whilst keeping the participant in full view of the investigator at all times. The curvature of the path varied slightly between sites. Four subjects (three not included in the study) performed reliability tests of the 30MW test between the sites. Subjects varied within 5% at each site and this was considered an acceptable level of variation given the need to be flexible with the location of the testing site.

The participant was instructed to walk as far as possible and safely for 30 minutes along the 200 m track, pivoting to turn at the end of each lap. Participants were permitted to rest in standing at any time if required and to resume walking as soon as able. Encouragement was given by the assessor after every lap. The assessor commenced timing as the participant stepped over the starting line and ceased at 30 minutes. At this point, the participant was instructed to maintain their position on the track while the assessor measured the distance of the partial lap with a trundle wheel. Testing was terminated if the participant reported that they were unable to continue and the distance covered in the abbreviated test was recorded as the distance covered in 30 minutes. To evaluate gait characteristics, the assessor recorded the number of laps walked, the number of steps and time taken during one lap in the initial five minutes and again during the last five minutes of the test, and the number and duration of any rests taken, noting the reasons for resting. From this, average cadence, average step length and total 30MW distance were calculated. To measure test-retest reliability of the 30MW test, participants repeated the test within two weeks of the first test using the same track.

#### Western Ontario and McMaster Universities Osteoarthritis Index VA3.1 (WOMAC)

The WOMAC index [25] was used to measure patient-reported pain and function. The pain and function subscales of the WOMAC index were scored using a visual analogue scale on a 10cm line for each question and a total was calculated for each subscale (worst score 50 for pain, 170 for function).

#### Physical activity questionnaire

The questions on physical activity from the National Physical Activity Survey [26], were used as the data provide a contemporary population-based comparison for our cohort. The total number of sessions and total duration of physical activity was calculated by summating reported sessions and duration of walking over 10 minutes, and moderate and vigorous activity.

### Statistical analysis

Descriptive statistics were calculated for continuous variables, and distribution was examined. Independent t-tests and Mann–Whitney U tests were used to compare subject characteristics and walking test variables as appropriate. Chi square test was used to compare difference in participants requiring rests between the groups. Paired t-tests were used compare the difference in average walking speed between 6MW and 30MW tests. Repeatability of the 30MW test was measured by calculating the intra-class coefficient (ICC) (two way random, absolute agreement). Correlation coefficients (Pearson’s or Spearman’s rank as appropriate) were computed between pairs of walk tests, and multiple linear regressions were used to determine the predictors of the 30MW test in the TKA cohort, respectively. Multiple linear regression modeling was used to establish predictive models for WOMAC function score and total time of physical activity in the TKA cohort. To compare the predictive value of the three walk tests, three models were generated for each dependent variable using gender, body mass index (BMI), and one of the walk tests as independent variables. Each model was limited to three independent variables due to the sample size. These variables were determined a priori, and were selected based on current literature [18,27-29]. Appropriate regression diagnostics were used in modeling. For all analyses, P < 0.05 was deemed as significant. Data from the first testing session of the 30MW test were used in the regression analyses.

## Results

Of the 118 patients who underwent TKA surgery at our institution between January and July 2008, 90 patients were randomly selected and screened. Based on exclusion criteria, 45 patients were eligible; 32 of these subsequently consented to participate. Forty-three healthy volunteers were also screened and recruited for the study. Subject characteristics, WOMAC pain and function scores, and weekly physical activity, are summarized in Table 1. In the data set, there were three missing values of TUG in the TKA cohort and one in the healthy cohort due to administration error. Whilst age, gender and height were similar between the TKA cohort and controls, all other measures were significantly different between the two groups. Performances in the TUG and 6MW tests were inferior amongst the TKA cohort compared to healthy controls (Table 2).

**Table 2:** TUG, 6MW and 30 MW test performance, mean (95% CI), of TKA recipients and controls

**Walk test**		**TKA**	**Controls**	**Between group comparison, P**
TUG (TKA : n = 29, Controls: n = 42)	Time, second (median, IQR)	7.4 (6.3 to 9.0)	5.0 (4.2 to 6.0)	<0.001
6MW test (TKA: n = 32; Controls n = 43)	Distance, meters	423.5 (380.1 to 466.8)	582.1 (559.9 to 604.2)	<0.001
	Speed, meters per second	1.18 (1.07 to 1.29)	1.61 (1.55 to 1.67)	<0.001
30MW test (TKA n = 32; Controls n = 43)	Distance, meters	2108 (1837 to 2381)	3086 (2981 to 3191)	<0.001
	Average step length, meters	0.68 (0.64 to 0.72)	0.81 (0.78 to 0.83)	<0.001
	Cadence, steps per minute	112 (107 to 117)	124 (120 to 128)	<0.001
	Speed, meters per second	1.17 (1.02 to 1.32)	1.71 (1.65 to 1.77)	<0.001

Abbreviations:

CI = confidence interval; IQR = Inter-quartile range; N = number; TKA = Total knee arthroplasty.

### Performance and repeatability of 30MW test

TKA recipients walked significantly shorter distances, with shorter step length and lower cadence than control subjects in 30 minutes (Table 2). No participants stopped to rest during the 6MW test. Significantly more participants amongst the TKA cohort required rests compared to controls during the 30MW test (12/32 TKA; 0/43 Controls; p<0.001). The number of rests ranged from one to more than ten occasions. TKA recipients reported factors which limited their performance included pain in the index knee (n = 5), back and other lower limb joint pain (n = 13), and poor aerobic fitness (n=10). Control subjects had a significantly faster walking speed in the 30MW test than the 6MW test (P < 0.001), while TKA recipients had very similar walking speeds (P = 0.83). Subgroup analysis found that for TKA participants who rested (n= 12), 6MW speed was 0.90 m/s compared to 30MW speed of 0.82 m/s (P = 0.08). For TKA participants who did not rest (n = 20), the difference between 6MW speed and 30MW speed was minor and did not quite reach significance (1.34 m/s vs 1.38 m/s, P = 0.05).

Both groups exerted similar effort during the test as measured using heart rate [TKA average time with over 65% age-predicted HR maximum 26.6 minutes (SD 7.9), mean HR 117 (SD 17) beats per minute; Control average time 29.8 (SD 0.5) minutes, mean HR 127 (SD 13) beats per minute]. Supplementary analysis revealed that the 30MW distance covered by the TKA cohort [mean 2360 m (SD 340), P<0.001] remained significantly lower than the age-matched group when TKA recipients with other conditions impairing mobility (such as symptomatic lower limb, back or respiratory disease) (n=13) were removed.

All control subjects and 12 TKA recipients performed a repeat test. TKA recipients were reluctant to perform a second test as they found the test to be difficult. For participants who performed a second test, excellent repeatability was observed in the distance walked as denoted by the very high ICCs obtained for both groups (Table 3). Effort was also consistent between the two tests, with 97% of the healthy cohort and 73% of TKA participants achieving above 65% age-predicted HR maximum for 25 minutes or more across both days. On average, distance walked was better in their second test, but the difference was only significant in controls (P = 0.006).

**Table 3:** Repeatability of average distance (95% confidence interval), metres, walked during the first and second 30MW test for TKA recipients and healthy controls

	**Test 1**	**Test 2**	**ICC**
TKA (n=12)	2130.7 (1542.9 to 2718.5)	2209.8 (1678.5 to 2741.1)	0.97 (0.90 to 0.99)
Controls (n=43)	3085.9 (2981.1 to 3190.7)	3138.7 (3029.7 to 3247.7)	0.96 (0.91 to 0.98)

Average time between tests was 8 days.

Abbreviations:

30MW = 30 minute walk; ICC = Intraclass Correlation Coefficient; N = number; TKA = Total knee arthroplasty.

### Correlations between 30MW test versus 6MW test or TUG, and predictors of 30MW test performance in TKA cohort

**Figure 1:**
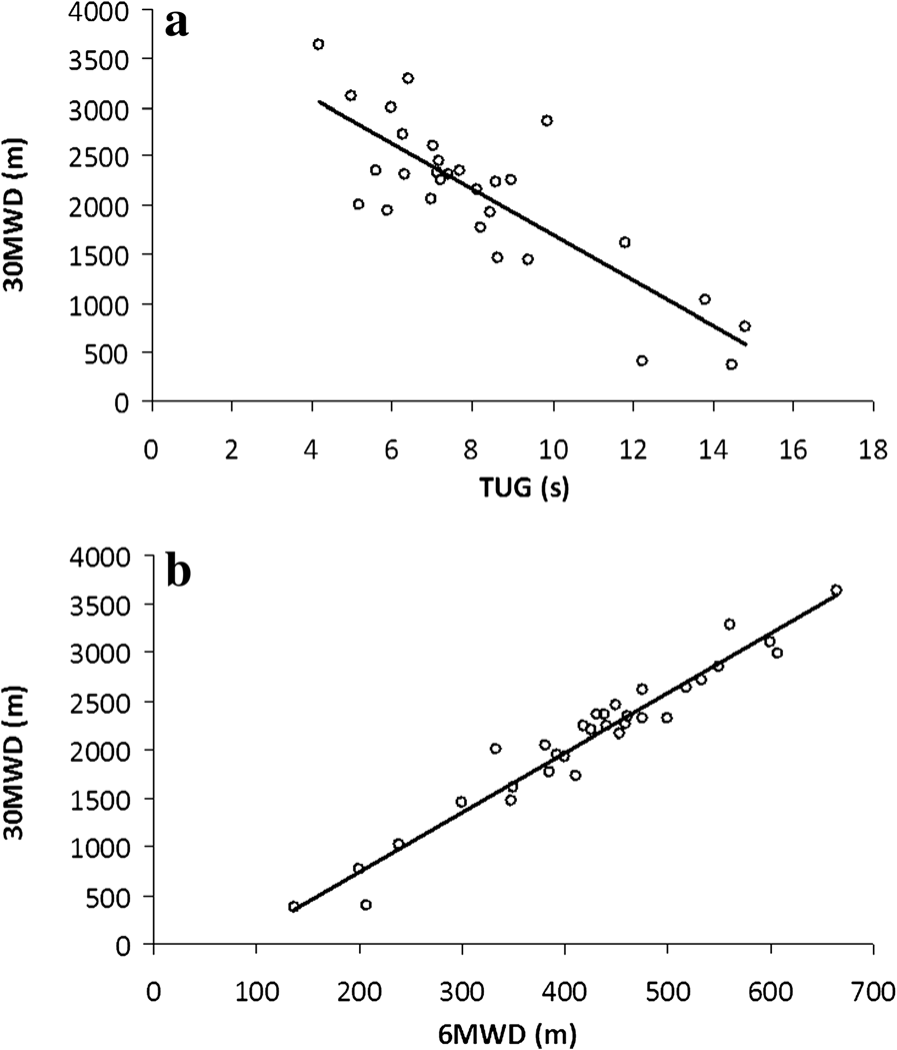
**Correlation between a) 30MWD** (n = 32) and **TUG** (n = 29, r = −0.82, P < 0.001; y = 6.10x – 472.01); **b) 30MWD and 6MWD** (n= 32, r = 0.97, P < 0.001; y = −230.3x + 3998.1). 30MWD = Thirty minute walk distance (meters); TUG = Timed up and go (seconds); 6MWD = Six minute walk distance (meters).

Scatter plots of 30MW distance versus TUG and 30MW distance versus 6MW distance for TKA patients are shown in Figure 1. Whilst the correlation between 30MW distance and TUG was strong (r = −0.82, P < 0.001), near-perfect correlation was found between 30MW distance and 6MW distance (r = 0.97, P < 0.001). Multiple regression modeling of 30MW distance using height, TUG and 6MW distance as variables found 6MW distance to be the only significant predictor (B 5.36, 95% CI 4.42 – 6.30, P < 0.001) of 30MW distance, with R^2^ of 0.96.

### Prediction of self-reported function and physical activities using walk tests

The significant predictor variables for self-reported function (WOMAC) and reported weekly duration of physical activity using multiple regression modes are shown in Table 4. Self-reported function was significantly predicted by each of the walk tests, with the TUG model being the most predictive, explaining 55% of the variance. In regression modeling of total duration of physical activity, TUG provided the only significant model, explaining 36% of the variance.

**Table 4:** Unstandardized regression coefficients, B (95% CI) on WOMAC function and total physical activity (TKA cohort)

**Independent Variable**	**Dependent Variable**			
	**WOMAC function**	**P**	**Physical Activity**	**P**
Model 1 (n = 28)	R^2^ = 0.55	<0.001	R^2^ = 0.36	0.01
BMI	0.67 (−1.27 to 2.61)	0.48	4.11 (−10.68 to 18.89)	0.57
Gender	−14.23 (−34.93 to 6.47)	0.17	45.06 (−112.70 to 202.77)	0.56
TUG	6.58 (3.16 to 10.0)	<0.001	−42.46 (−68.52 to −16.40)	0.003
Model 2 (n = 32)	R^2^ = 0.34	0.01	R^2^ = 0.16	0.18
BMI	0.40 (−1.83 to 2.62)	0.72	3.14 (−12.64 to 18.92)	0.69
Gender	−15.14 (−38.30 to 8.03)	0.19	14.23 (−150.10 to 178.51)	0.86
6MW distance	−0.10 (−0.20 to −0.01)	0.04	0.67 (0.00 to 1.35)	0.05
Model 3 (n = 32)	R^2^ = 0.34	0.01	R^2^ = 0.16	0.19
BMI	0.33 (−1.90 to 2.57)	0.76	3.44 (−12.47 to 19.34)	0.66
Gender	−16.39 (−39.17 to 6.40)	0.15	23.39 (−138.89 to 185.64)	0.77
30MW distance	−0.02 (−0.03 to −0.001)	0.04	0.10 (−0.02 to 0.21)	0.05

Abbreviations:

6MW = Six-minute walk; 30MW = 30-minute walk; BMI = Body mass index; CI = Confidence interval; N = number; TKA = Total knee arthroplasty; TUG = Timed Up and Go; WOMAC = Western Ontario and McMaster Universities Osteoarthritis Index.

## Discussion

This study deepens our understanding of walking ability of individuals one year after TKA surgery. The prevailing norm in this area is to use short duration walk tests to evaluate functional mobility after surgery [1,2,6-8]. It is unclear whether such tests truly reflect ambulatory capacity more typical of daily requirements in this population. This study contributes to the existing literature on both ambulation capacity after TKA and tests used to measure capacity. For the first time, we have data which provide comparisons between age-matched norms and TKA recipients in terms of distance walked over a 30 minute period. Likewise, we now have confirmation that the 6MW test is an excellent predictor of longer duration ambulation after TKA. Consequently, our study provides long overdue construct validity for the use of 6MW test as a measure of functional ambulation in TKA recipients [30]. Because of the close relationship between the 6MW and 30MW tests, contrary to our expectations, the 30MW test did not demonstrate greater capacity to differentiate between individuals nor have stronger correlations with patient-reported outcomes compared to the 6MW test. As such, an extended walk test is no more useful as a research tool than the shorter walk tests in this population and yet is more arduous.

We observed that the 30-minute walking capacity of TKA recipients at one year after surgery remains significantly inferior to their healthy counterparts. Given the similar effort observed during the 30MW test between TKA recipients and controls and the subjective reporting by participants, the inferior performance is likely explained by the presence of a prosthetic joint and pain in the index joint, as well as other factors shown to influence recovery such as the presence of significant co-morbidities [31], severe other joint disease [18], and obesity [18,28]. Lower physical activity levels reported by the TKA cohort, which are a likely manifestation of the co-morbidities, joint disease and obesity associated with patients requiring TKA, may also be a contributing factor to their poorer performance. Despite their limitations, all TKA recipients completed the test without adverse events, indicating that though the test is arduous, it appears to be safe. The 30MW test was observed to be highly repeatable. The healthy cohort walked significantly further by 50 m in the second test. This difference may be due to a practice effect, which has been described by some authors in the 6MW test [16]. It is likely that a significant increase was not seen in the TKA cohort because there was greater variability in their performance, as indicated by a larger standard deviation of the difference between the first and second test (TKA SD 215 m vs Controls SD 93 m). The reluctance by patients to repeat the test reflects that it may not be embraced in this population, but this does not alter the new insights gained about ambulatory capacity, the various associations between the walk tests, or between the walk tests and patient-reported outcomes.

The observed residual impairment in mobility amongst our TKA cohort is further supported by comparisons between their 6MW test performance with published standard values of healthy elderly populations. Using the reference equation published by Troosters et al. [16], our healthy cohort performed very similarly to their predicted distance (582 m vs predicted 576 m), while the performance of our TKA cohort was much lower than their predicted distance (424 m vs predicted 538 m). As this reference equation takes into account age, gender, height and weight (the constituents of BMI), it demonstrates that the higher BMI of the TKA cohort only makes a minor contribution to the large difference in walking performance compared to the healthy cohort. Using reference equations published by Enright et al. [32], which have been considered by some to underestimate normal walking distance [10,33], the performance of our TKA cohort was still inferior, with the predicted distance for TKA group of 464 m.

The near-perfect correlation between the 6MW and 30MW tests and the high predictability of 30MW distance by 6MW distance were unexpected. This is because participants were instructed to walk as far as possible but safely, and as such we expected that some participants would not be able to maintain the same speed in both tests, presuming a comparatively poor fitness level in the cohort. We observed that the healthy cohort walked faster in the longer test. This may be due to less frequent deceleration and turning associated with the longer lap in the 30MW test. In contrast, the walking speeds of TKA recipients were identical in both tests. The gain in walk speed associated with a longer track was not seen in the TKA cohort, suggesting that the 30MW test was indeed more fatiguing for this cohort. For some participants, the overall walk speed was reduced as a result of the rests taken during the 30MW test and these people demonstrated the slowest speeds in the 6MW test. For those who did not rest, there was a trend for a slightly faster speed but this did not quite reach statistical significance. Consequently, whilst fatigue likely did become a factor in the longer test, its effects were small, and those most affected were those who performed worst in the 6MW test anyway. Thus the prediction of the 30MWD and 6MWD was high. The knowledge that a strong correlation exists between the 6MW and 30MW tests is useful both in clinical practice and research, as it means that the 6MW test is a suitable tool to evaluate interventions aimed at improving longer duration ambulation; specifically, the 6MW test will predict how far a TKA recipient can walk over 30 minutes with acceptable accuracy.

All three walk tests moderately predicted self-reported function. The 6MW and 30MW tests produced similar models for reasons described above, with TUG producing the strongest model. A possible explanation for the latter is that, unlike the 30MW and 6MW tests, TUG performance is a combination of walking ability, lower limb strength and balance owing to the sit-to-stand component, and is based on activities similar to items included in the WOMAC questionnaire. Others have found similar, modest relationships between measured ambulation and self-reported function. Rossi et al. [11] found moderate correlation between TUG and perceived function amongst participants 17 months after TKA. Another study examining short-term recovery also found only moderate correlation between self-reported function and performance based outcomes [12]. Our results, based on a cohort 1 to 1.5 years post-surgery, add to the growing body of literature justifying the collection of both performance-based and self-reported measures in assessing longer-term recovery after TKA.

Physical activity levels reported by the TKA cohort were significantly lower than the control group. The average time spent performing physical activities by TKA recipients (202 minutes) was also lower than reported in a similarly aged population-based sample (255 minutes) using the same survey [26], whilst the activity levels of our healthy cohort (mean 504 min) were much greater. Whilst these comparisons with population-based data appear to corroborate the representativeness of both our patient and healthy cohorts in terms of self-reported physical activity, we remain unclear as to the relationship between self-reported physical activity and measured ambulation. TUG was a significant, but weak predictor of self-reported physical activity in our TKA cohort. A possible explanation for the weak relationship is that physical activity is determined by other factors such as self–efficacy [34], personality, knowledge, beliefs and social support [35] and not just physical mobility. Alternatively, patient-reported physical activity may not reliably measure actual physical activity [36]. It is not known how accurately the survey questions we used correlate with actual performance. We used questions used by a large Australian population-based study - the National Physical Activity Survey [26] - for the purposes of having a population-based comparison. The trade-off for this approach is the lack of certainty with which these questions do reliably reflect actual physical activity.

By virtue of our design, this study also describes for the first time the spatio-temporal gait characteristics of TKA recipients under natural environmental conditions, as gait analysis in this population has mostly been conducted from snap-shots created under laboratory conditions [37]. Whilst the shorter step length and lower cadence observed compared to age-matched controls are consistent with published data [38-40], the lower cadence in our TKA recipients, compared to that observed during fast walking in the laboratory amongst participants with similar characteristics [37], may reflect the use of different strategies to achieve fast walking in different environments. Further study of gait parameters in natural walking environments may give greater insight into the persistent walking limitations in the TKA population.

A limitation to the study is that the results are limited to the TKA population one year post-surgery. A minor limitation of the study is the sample size of the TKA cohort as it necessarily restricted the number of variables included in the regression modeling. A larger sample would have allowed the inclusion of other potential predictors, such as age and co-morbidity which are known predictors of the shorter walk tests [41-43]. This notwithstanding, as 6MW distance was such a strong predictor of the 30MW distance, it is unlikely that inclusion of other variables would change the definitive model substantially. Furthermore, factors such as age, gender and BMI are accounted for to some degree by their association with 6MW distance [41,42]. A potential limitation of the study was that our TKA cohort included TKA recipients with and without the common mobility-limiting co-morbidities. A sample which excluded patients with significant co-morbidity would have enabled a more straight-forward comparison between age-matched peers and TKA recipients, and eliminate the variability in walk tests due to factors other than the TKA surgery. However, such a sample would not be representative of typical TKA cohorts. We note that subgroup analyses of TKA recipients with minimum co-morbidities still demonstrated significantly inferior performance compared to healthy controls. Not randomizing the order of testing to eliminate the effect of fatigue during testing could be viewed as another potential limitation in this study. However, the amount of time required after the 30MW test to ensure sufficient rest made this impractical. Furthermore, it was unlikely that fatigue affected testing because amongst the TKA cohort, in whom fatigue would have a larger potential impact, those who returned for a repeat 30MW test (which did not follow a 6MW test) did not perform significantly better in the second test.

## Conclusions

The 30MW test highlights the persistent subnormal mobility seen in this cohort, its utility, however, is lacking as it does not provide greater predictive value for patient-reported outcomes than the shorter walk tests and the test appears particularly arduous. As the 30 MW test is almost completely predicted by the 6MW test, this study demonstrates that the 6MW test is a robust measure of functional mobility in long-term recovery amongst TKA recipients. Our findings reinforce the use of performance-based tests such as the 6MW and TUG tests together with self-report outcomes to evaluate recovery of functional mobility.

## Abbreviations

6MW: Six-minute walk; 30MW: 30-minute walk; BMI: Body mass index; HR: Heart Rate; ICC: Intra-class correlation coefficient; TKA: Total knee arthroplasty; TUG: Timed-Up-and-Go; WOMAC: Western Ontario and McMaster Universities Osteoarthritis Index.

## Competing interests

The authors declare that they have no competing interests.

## Authors’ contributions

VK contributed to the conception and design of the study; acquisition, analysis and interpretation of data; and drafting of the manuscript. JN contributed to the conception and design of the study; acquisition, analysis and interpretation of data; and drafting the manuscript. IH contributed to the conception and design of the study; interpretation of data; and revision of the manuscript. JC contributed to the conception and design of the study; analysis and interpretation of data; and revision of the manuscript. AY contributed to the analysis and interpretation of data; and revision of the manuscript. All authors read and approved the final manuscript.

## Pre-publication history

The pre-publication history for this paper can be accessed here:

http://www.biomedcentral.com/1471-2474/14/145/prepub
